# Visual function and retinal changes after voretigene neparvovec treatment in children with biallelic *RPE65*-related inherited retinal dystrophy

**DOI:** 10.1038/s41598-022-22180-6

**Published:** 2022-10-21

**Authors:** Francesco Testa, Paolo Melillo, Valentina Di Iorio, Claudio Iovino, Francesco Farinaro, Marianthi Karali, Sandro Banfi, Settimio Rossi, Michele Della Corte, Francesca Simonelli

**Affiliations:** 1 Eye Clinic, Multidisciplinary Department of Medical, Surgical and Dental Sciences, University of Campania Luigi Vanvitelli, Via S. Pansini 5, Building 15, 80131 Naples, Italy; 2 Medical Genetics, Department of Precision Medicine, University of Campania Luigi Vanvitelli, via Luigi De Crecchio 7, 80138 Naples, Italy; 3 Telethon Institute of Genetics and Medicine, via Campi Flegrei 34, 80078 Pozzuoli, Italy

**Keywords:** Tomography, Paediatric research, Retinal diseases

## Abstract

To report quantitative retinal changes assessed by spectral-domain optical coherence tomography (SD-OCT) in children treated with voretigene neparvovec (VN) at a single center in Italy. Retrospective review of six consecutive pediatric patients with biallelic *RPE65*-related dystrophy treated bilaterally with VN. SD-OCT scans were analyzed to extract Early Treatment Diabetic Retinopathy Study (ETDRS) thickness maps of the whole retina and the outer nuclear layer (ONL). Changes in visual function were assessed by best-corrected visual acuity (BCVA) and retinal morphology at Days 30/45 and 180. BCVA significantly improved at Day 30/45 and 6 months (both *P* < 0.001). Central foveal retinal thickness and central foveal ONL thickness tended to increase (6.4 ± 19.2 µm; *P* = 0.080 and 3.42 ± 7.68 µm; *P* = 0.091, respectively). ONL thickness of the internal ETDRS-ring significantly increased at day 30/45 (4.7 ± 8.4 µm; *P* < 0.001) and day 180 (5.0 ± 5.7 µm; *P* = 0.009). Intra-operative foveal detachment was not associated with a higher function gain in terms of BCVA, but with a mild thinning of foveal ONL after treatment. The improvement of BCVA and thickening of the ONL layer suggest that improvement of visual acuity could be related to partial recovery of retinal morphology in the perifoveal ring.

Biallelic mutations in the *RPE65* gene cause an early-onset, severe, rod-mediated hereditary retinal dystrophy, which progresses to low vision or blindness^1–3^.

A phase 1/2 clinical trial and a subsequent randomized controlled phase 3 trial showed the safety and efficacy of a unilateral injection of the recombinant adenoviral vector voretigene neparvovec (VN) for the treatment of biallelic *RPE65* mutation-related inherited retinal dystrophy (*RPE65*-IRD)^4,5^. Based on the improvements in visual function after treatment with VN in these trials^4,5^ (particularly in terms of navigational ability under dim lighting conditions, light sensitivity, and visual field), VN was approved by the US Food and Drug Administration (FDA) and the European Medicines Agency (EMA) for the treatment of patients with biallelic *RPE65*-IRD^6,7^.

While changes in visual function after treatment with VN have been described both in the clinical trial reports^4,5,8,9^ and in real-life case series^10–12^, limited quantitative morphological evaluations are available for patients treated with VN. In fact, in the phase 1 and phase 3 trials^4,5,8,9^, spectral-domain optical coherence tomography (SD-OCT), specifically central retinal thickness, was used only to evaluate patient eligibility for inclusion and to assess the safety of the treatment.

Only two recent case series have investigated quantitative retinal changes in patients treated with VN^10,12^, but their morphological assessments were focused on central foveal retinal thickness.

Imaging using SD-OCT has enabled direct measurements of outer nuclear layer (ONL) thickness in animal models to monitor the effects of therapeutic interventions^13–15^. As the ONL contains the nuclei of cone and rod photoreceptors^16^, ONL thickness may be more representative of the effects of gene therapy on photoreceptor degeneration than retinal thickness as a whole. Despite evidence from pre-clinical studies, few reports analyzed ONL thickness in biallelic *RPE65* mutation-related dystrophy after treatment^17,18^. However, in these studies patients were treated with a different gene therapy product, rAAV2-RPE65. Furthermore, while the report of the phase 3 clinical trial showed amelioration of rod-mediated visual function with a statistically significant improvement of the related outcomes, improvements of visual acuity after VN treatment were not statistically significant and there was high variability among subjects^8^.

Since we previously observed a clinically significant improvement of visual acuity in our cohort of treated pediatric patients^19^, we postulated that analysis of the ONL could be useful to better understand the effects of treatment with VN and to determine the improvement in visual acuity. Therefore, in the current study we aimed to investigate quantitative retinal changes assessed by SD-OCT, focusing on measurements of the thickness of the whole retina and of the ONL in a cohort of pediatric subjects treated with VN.

## Materials and methods

This retrospective study includes a comprehensive review of all consecutive patients with biallelic *RPE65*-IRD treated with VN after EMA approval at the Centre for Rare Ocular Disease of the University of Campania Luigi Vanvitelli, Naples, Italy (formerly known as the Centre for Inherited Retinal Dystrophies of the Second University of Naples). All patients and/or their legal guardians/parents gave their informed consent to participate in the post-approval study by Novartis, the VN licensor in the European Union (EU) covering the Centres of Excellence administering VN in the EU and countries other than the United States, and all procedures adhered to the tenets of the Declaration of Helsinki and all relevant local regulations. Ethics approval was obtained from the institutional review board of the University of Campania Luigi Vanvitelli.

The pediatric patients received VN at our Centre between December 1, 2019, and July 1, 2021. In particular, our cohort of treated pediatric patients included 10 subjects (4 males and 6 females) treated with VN at a mean age of 10.8 ± 3.5 years (range 7–17 years). All patients satisfied the criteria for treatment with VN (e.g., central retinal thickness > 100 µm), but four patients showed nystagmus before treatment, which did not enable the acquisition of baseline volumetric SD-OCT scans of a satisfactory quality. Therefore, in this study, we included only six subjects with volumetric SD-OCT scans suitable to allow reliable layer segmentation analysis of the macular area before treatment with VN. Consequently, the study sample consists of 2 males and 4 females treated with VN at a mean age of 10.2 ± 3.1 years (range 7–16 years). Their main demographic and genetic features are summarized in Table 1. Segregation analysis was performed in the unaffected parents of all six probands, confirming that the variants identified in each patient were present in trans (i.e., on the maternal and paternal allele).

**Table 1:** Main demographic and genetic features of the study sample at baseline.

ID	Age (years)	Gender	Date of injection		BCVA (logMAR)		*RPE65*-associated mutations	
			RE	LE	RE	LE	Allele 1	Allele 2
1^†^	9	Male	11-Dec-19	27-Nov-19	0.7	0.8	c.938A > G (p.His313Arg)	c.1445A > T (p.Asp482Val)
3	9	Female	7-Apr-21	24-Mar-21	0.62	0.7	c.1102T > C (p.Tyr368His)	c.1229C > A (p.Ser410*)
4	7	Female	7-Apr-21	24-Mar-21	0.6	0.6	c.1102T > C (p.Tyr368His)	c.1229C > A (p.Ser410*)
5	11	Male	31-Mar-21	14-Apr-21	0.74	0.7	c.370C > T (p.Arg124*)	c.1543C > T (p.Arg515Trp)
6	9	Female	14-Apr-21	31-Mar-21	0.62	0.8	c.1543C > T (p.Arg515Trp)	c.1555G > T (p.Glu519*)
11	16	Female	16-Jun-21	1-Jul-21	0.82	0.7	c.65T > C (p.Leu22Pro)	c.10C > T (p.Gln4*)

All nucleotide positions refer to the transcript NM_000329.

*BCVA* best-corrected visual acuity, *RE* right eye, *LE* left eye.

^†^Follow-up data related to patient 1 has been already reported in Testa et al. (2021).

A single surgeon (MDC) performed subretinal VN therapy according to the general guidelines of the recommended protocol reported by Russell et al.^5^, as previously described^19^. Briefly, the surgical technique was OCT-guided 25-gauge vitrectomy (Alcon CONSTELLATION^®^ Vision System, Geneva, Switzerland). Sclerotomies were performed at 3.5 mm from the limbus. Following standard core vitrectomy, posterior vitreous detachment was induced with preservative-free triamcinolone acetonide. Next, single subretinal injection of VN was performed along the upper vascular arcades, avoiding vascular structures and areas of atrophy, at least 2 mm away from the foveal center, using a 25/38-gauge needle (Cannula PolyTip 25 g/38 g, MedOne Surgical, Inc., FL, USA). Although the injection strategy was the same in all the eyes, since the bleb direction is unpredictable in patients with inherited retinal dystrophies^20^, the bleb included the fovea only in some cases, inducing an intra-operative foveal detachment.

No intra-operative surgical complications occurred and none of the patients in our study experienced any of the previously reported adverse events (e.g., retinal tear, macular hole, endophthalmitis, or cataract). Nevertheless, three patients (ID 3, 4, and 11) developed new areas of focal retinal atrophy—exceeding the retinotomy site—in retinal midperiphery (beyond vascular arcades) identified by evident loss of the RPE (i.e., a variable degree of translucency with a detectable choroidal vasculature), which were observed in both eyes at the 6-month post-treatment timepoint.

In all patients, the worst-seeing eye was treated first; the contralateral was treated about 15 days later. Patients received 1 mg/kg per day (up to 40 mg/day) of oral prednisone for 7 days, starting 3 days before the first injection. Prednisone was tapered (0.5 mg/kg per day, up to 20 mg/day) for the following 7 days or until 3 days before the injection of the second eye, when the steroid regimen was repeated.

For the purpose of the current study, the effects of VN treatment on visual function were assessed using best-corrected visual acuity (BCVA) tests both before and after treatment (approximately 45 days after the treatment of the first eye, which corresponded to about 30 days post-treatment of the second eye), as well as at about 180 days after the treatment of both eyes. Changes in retinal morphology were evaluated by SD-OCT at the same time points.

A standard protocol based on Early Treatment Diabetic Retinopathy Study (ETDRS) charts was used to measure BCVA. Letter scores were converted to the logarithm of the minimum angle of resolution (logMAR).

SD-OCT images of the posterior pole were acquired with the Spectralis OCT plus with blue peak (Heidelberg Engineering, Heidelberg, Germany), obtaining a dense 20° × 15° volume scan centered onto the fovea with 19 horizontal B-Scan (interscan distance of 263 μm) and a minimum of five automatic real-time tracking frames each. SD-OCT images at the different time points were acquired on exactly the same location of the fundus via the AutoRescan™ function provided by the device (also known as follow-up examination) in order to achieve reliable measurement. Automatic segmentation was carried out through the inbuilt tool (Eye Explorer version 1.9.10.0). The tool provided ETDRS thickness maps for the whole retina (i.e., from the inner limiting membrane to the Bruch’s membrane) and for each retinal layer, including ONL (i.e., from the outer plexiform layer to the external limiting membrane). After performing automatic segmentation, a skilled operator inspected each B-scan to identify and correct eventual segmentation artifacts within the ETDRS area. Thickness values from the central foveal ETDRS subfield and the average among the four ETDRS subfields of the internal ring were then collected for the whole retina and for the ONL. Normative values for thickness were computed on a sample of 10 pediatric patients (mean age: 12.0 ± 5.5 years) without any ocular or systemic manifest disease and a BCVA of 20/20. Furthermore, the foveal ellipsoid zone band (EZ band) and the external limiting membrane (ELM) were graded in the foveal B-scan as intact, disrupted (discontinuous or granular appearance), absent^21^. Grading was undertaken independently by two expert graders and, in case of disagreement, was adjudicated by a third grader.

### Statistical analysis

The statistical analysis was performed using the SPSS Statistics platform (IBM, Armonk, New York, version 21.0.0.0). Descriptive statistics (e.g., mean ± standard deviation for continuous variables) were calculated using both eyes of each patient. A generalized estimating equation (GEE) was applied for statistical comparison by adopting an appropriate covariance structure, as previously described by Glynn and Rosner^22^. The method can accommodate inter-eye correlation between the two eyes of the same subject at a given visit, in addition to the longitudinal correlation between values of the same eye followed over time. The follow-up values were compared to baseline values of the same eyes at the first post-treatment time point (i.e., about 45 days, or 30 days after treatment of the first and second eye, respectively) and the last one (i.e., about 180 days after treatment) values. Functional and morphological outcomes were also analyzed to compare results in eyes with and without intra-operative foveal detachment.

## Results

The mean baseline BCVA prior to VN therapy was 0.70 ± 0.08 logMAR (corresponding to an ETDRS score of 50 and a Snellen equivalent of 20/100). At the Day 30 / Day 45 post-treatment, a significant (*P* < 0.001) mean improvement of 0.15 ± 0.06 logMAR (corresponding to 8 ETDRS letters) was recorded. As summarized in Table 2, a further improvement was observed 6 months after treatment with a significant change compared to baseline (-0.20 ± 0.07 logMAR; *P* < 0.001). In particular, six eyes (50%) showed an improvement of one ETDRS line, whereas BCVA in the remaining six eyes (50%) improved by two ETDRS lines.

**Table 2:** Change of best-corrected visual acuity over the 6-month follow-up period.

Time-point	ETDRS letter scores*	Change from baseline*	*P* value^†^
Baseline	0.70 ± 0.08		
Day 30/Day 45	0.55 ± 0.06	− 0.15 ± 0.06	**< 0.001**
Day 180	0.50 ± 0.09	− 0.20 ± 0.07	**< 0.001**

*ETDRS* early treatment diabetic retinopathy study.

*Data reported as mean ± standard deviation.

^†^*p*-values are obtained by generalized estimating equation regression models; statistically significant p-values are shown in bold.

**Figure 1:**
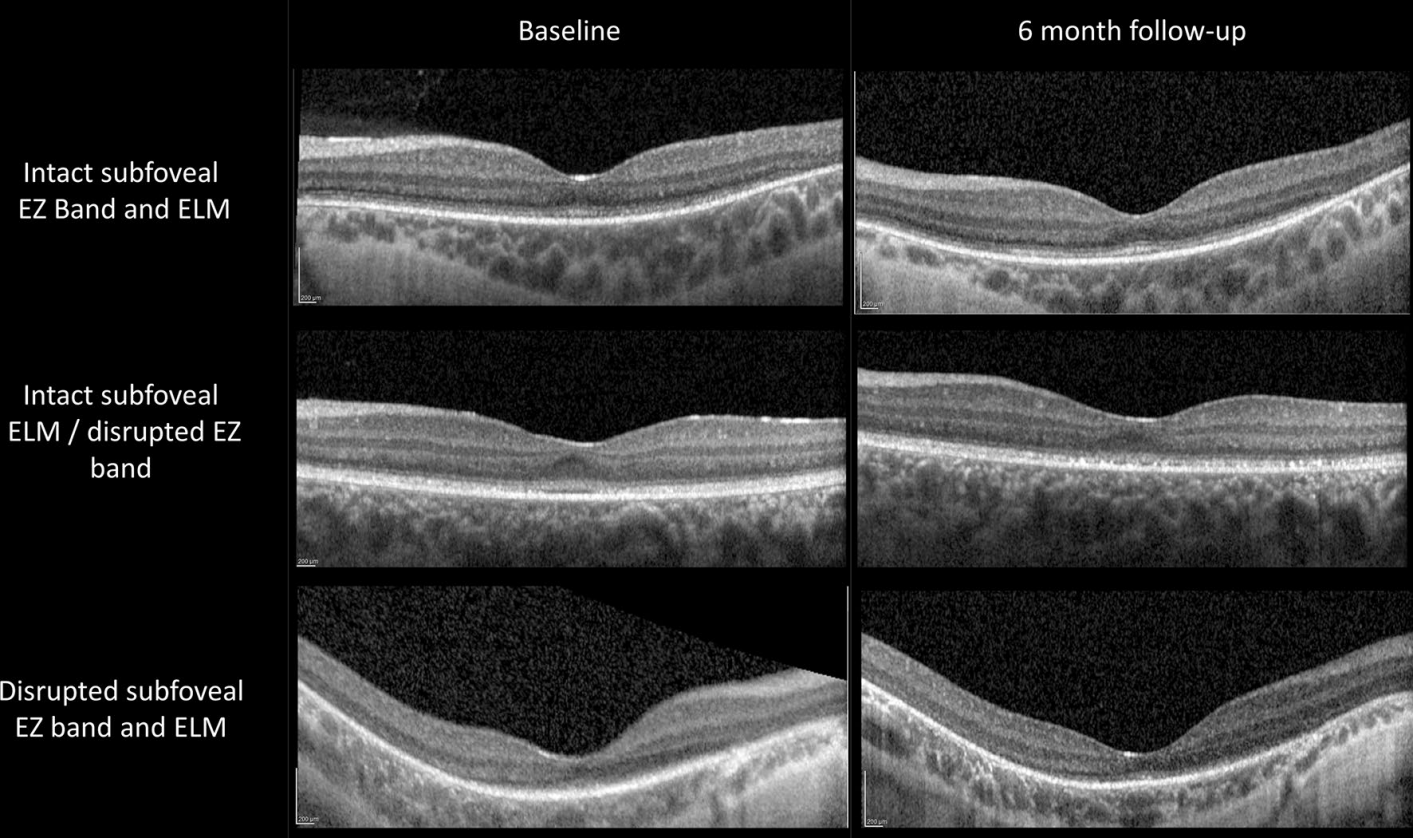
Spectral-domain optical coherence tomography (SD-OCT) scans at the baseline and at the six-month follow-up in three selected cases, showing no significant changes in qualitative evaluation after treatment with voretigene neparvovec (VN). ELM = external limiting membrane; EZ = ellipsoid zone.

Qualitative evaluation of foveal SD-OCT scans at the baseline showed a disrupted subfoveal EZ band in all except one eye, in which the subfoveal EZ band was intact. The ELM band was graded as disrupted in all but two eyes, which had intact ELM bands. During follow-up, we observed no significant change in the grading of the EZ and the ELM bands. Figure 1 shows SD-OCT scans at the baseline and at the six-month follow-up in three selected cases. Thickness analysis at the baseline showed reduced central foveal retinal thickness (225.9 ± 28.2 µm) compared to age-matched healthy eyes (266.3 ± 11.0 µm; range: 256–286 µm). Similarly, central foveal ONL thickness was reduced (54.2 ± 14 µm, range: 36–75 µm; age-matched healthy eyes: 90.1 ± 11.2 µm, range: 77–112 µm). Furthermore, the mean ONL thickness of the internal ETDRS ring (1–3 mm from the fovea) was reduced in all eyes (37.7 ± 11.0 µm; range: 19–56.5 µm; age-matched healthy eyes: 68.1 ± 6.0 µm; range: 58–79 µm).

**Figure 2:**
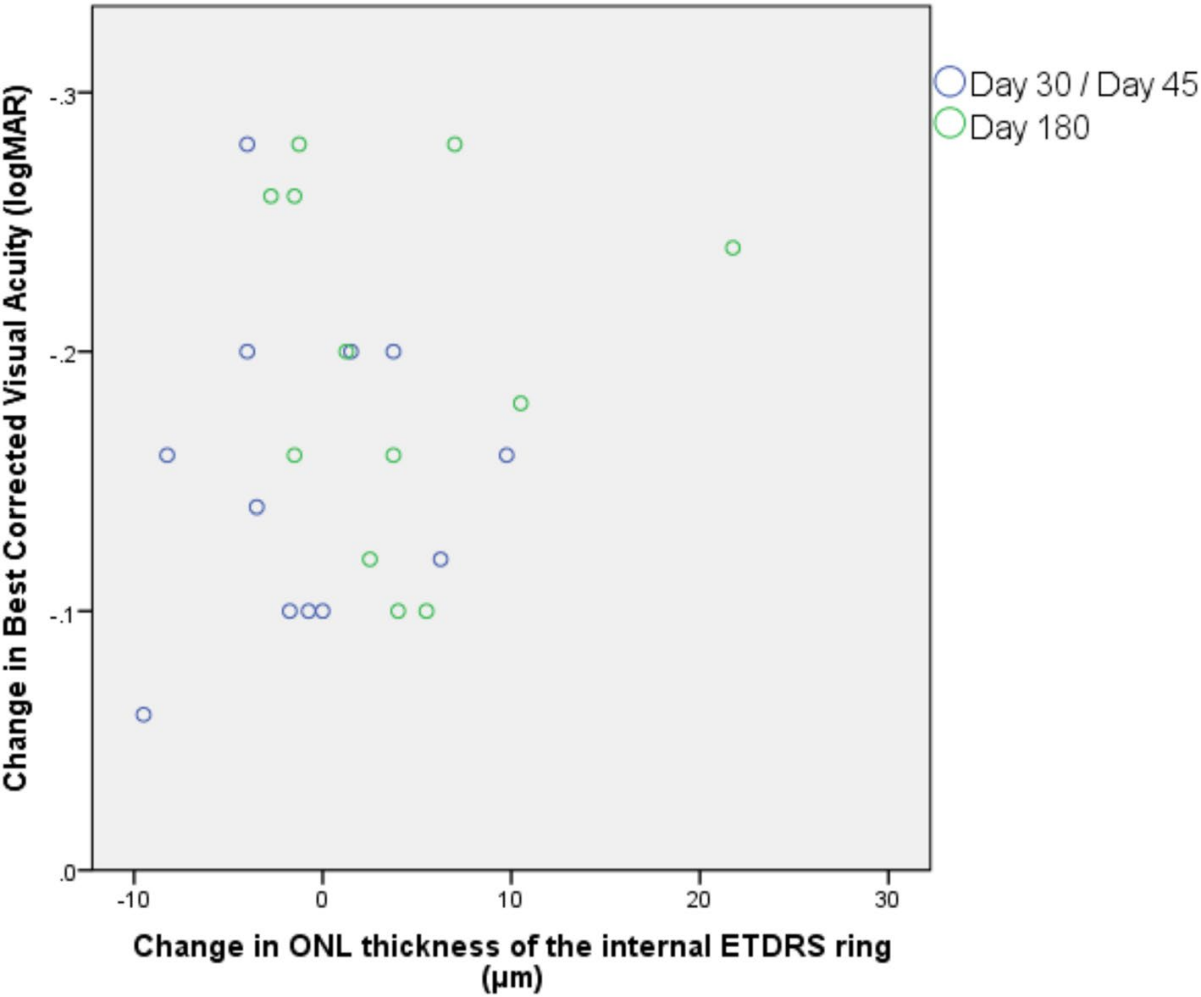
Scatterplot of changes over the 6-month follow-up in best-corrected visual acuity (BCVA) versus changes in outer nuclear layer (ONL) thickness of the internal Early Treatment Diabetic Retinopathy Study (ETDRS) ring.

As summarized in Table 3, at day 180, we observed a trend toward increased central foveal retinal thickness (6.4 ± 19.2 µm; *P* = 0.080) and central foveal ONL thickness (3.42 ± 7.68 µm; *P* = 0.091), whereas a statistically significant increase of ONL thickness of the internal ETDRS ring was observed both at the day 30/45 (4.7 ± 8.4 µm; *P* < 0.001) and day 180 (5.0 ± 5.7 µm; *P* = 0.009) time points. Moreover, analyzing the relationship between BCVA gain and change in ONL thickness at the six-month time-point compared to baseline, we observed that a higher improvement in BCVA was significantly associated with a higher increase in ONL thickness in the internal ETDRS ring, as shown in Fig. 2 (β = − 0.001; *P* = 0.010).

**Table 3:** Change of the selected spectral-domain optical coherence tomography parameters over the 6-month follow-up period.

Parameter	Mean thickness*	Mean change*	*P* value^†^
**Central foveal retinal thickness (µm)**			
Baseline	225.9 ± 28.2		
Day 30/Day 45	228.5 ± 31.3	2.4 ± 10.2	0.820
Day 180	232.3 ± 43.4	6.4 ± 19.2	0.080
**Central foveal ONL thickness (µm)**			
Baseline	54.3 ± 13.9		
Day 30/Day 45	53.3 ± 10.7	− 1.1 ± 8.5	0.815
Day 180	57.7 ± 15.7	3.4 ± 7.7	0.091
**ONL thickness of the internal ETDRS ring (µm)**			
Baseline	37.7 ± 11.4		
Day 30/Day 45	42.4 ± 8.6	4.7 ± 8.4	**< 0.001**
Day 180	42.7 ± 9.8	5.0 ± 5.7	**0.009**

*ETDRS* early treatment diabetic retinopathy study, *OCT* optical coherence tomography (OCT), *ONL* outer nuclear layer.

*Data reported as mean ± standard deviation.

^†^*p*-values are obtained by generalized estimating equation regression models; statistically significant *p*-values are shown in bold.

**Figure 3:**
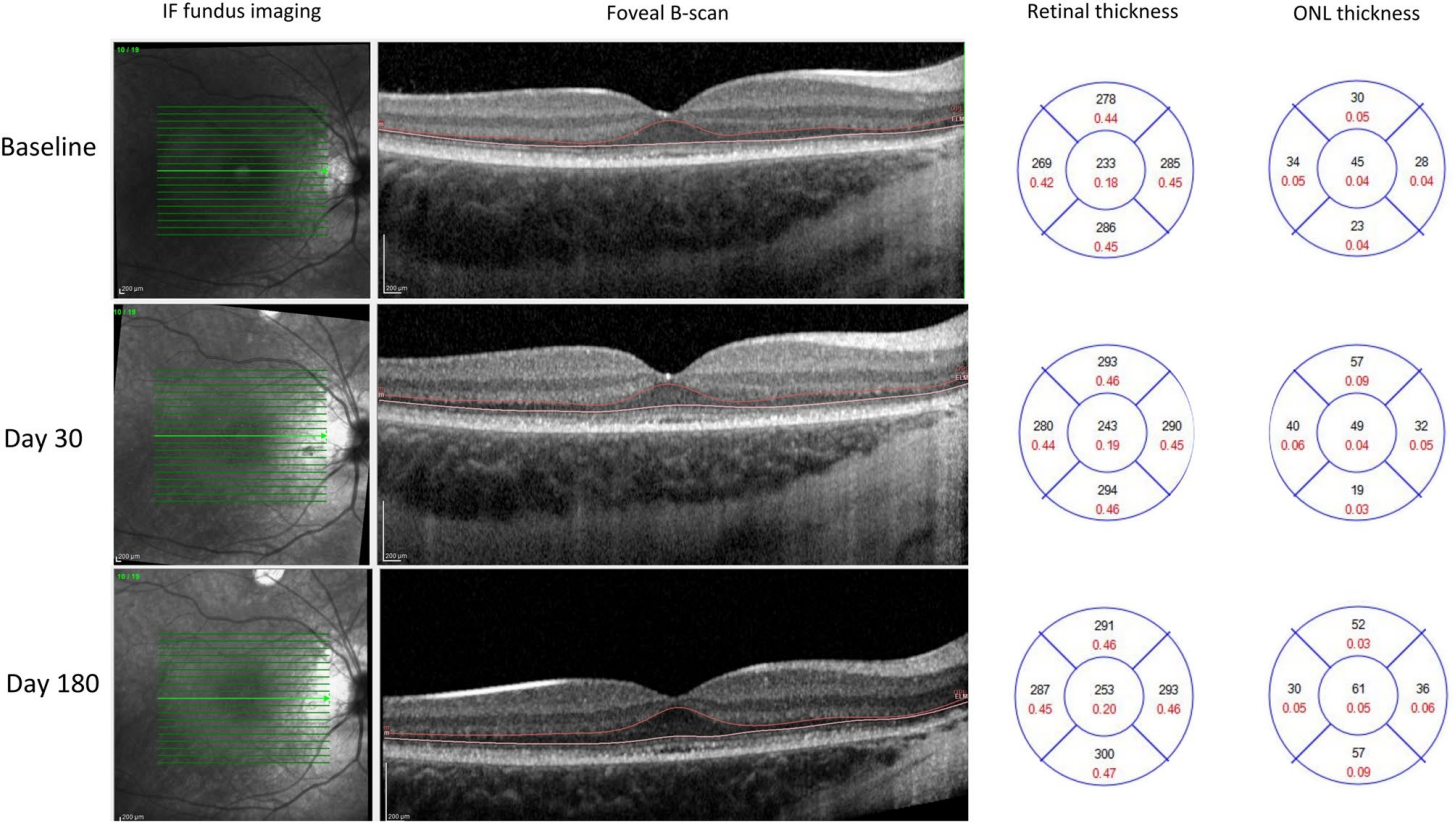
Retinal and outer nuclear layer (ONL) thickness analysis in the right eye of patient 6 not undergoing intra-operative foveal detachment, showing thickening of the ONL after treatment with voretigene neparvovec (VN).

**Figure 4:**
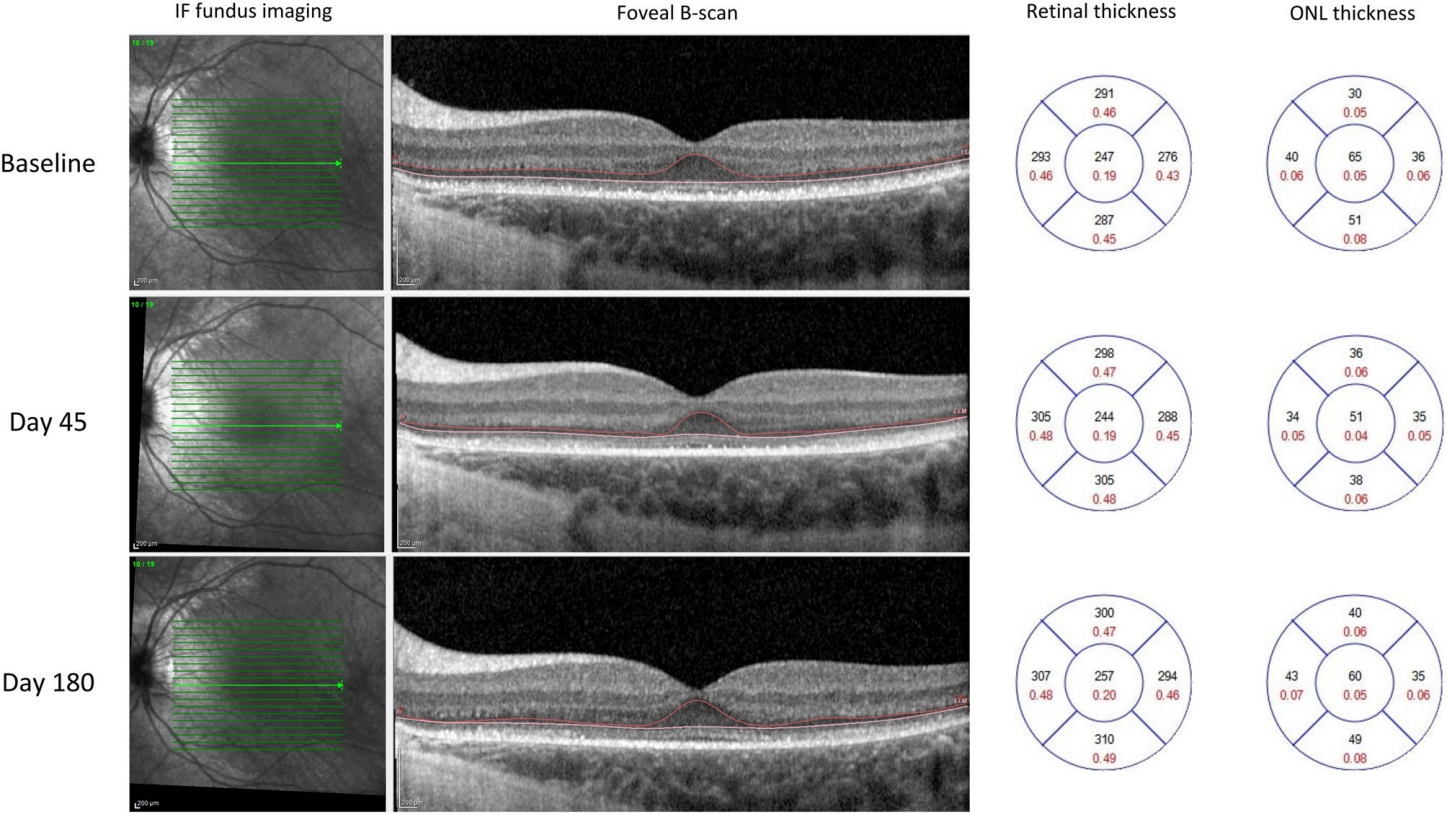
Retinal and outer nuclear layer (ONL) thickness analysis in the left eye of patient 6 undergoing intra-operative foveal detachment, showing a mild thinning of central subfoveal ONL after treatment with voretigene neparvovec (VN).

Table 4 compares the BCVA and SD-OCT analyzed parameters in the eyes with and without intra-operative foveal detachment. In particular, BCVA improved in both subgroups with no significant between-group difference. On average, central foveal retinal thickness mildly decreased in eyes undergoing intra-operative foveal detachment and increased in those without, even if these differences were not statistically significant (*P* = 0.054). We observed a significant difference in central foveal ONL thickness (*P* = 0.022) with a mild thinning in eyes undergoing intra-operative foveal detachment and a thickening in those without foveal detachment. No significant differences were observed in the ONL thickness of the internal ETDRS ring. Figure 3 showed thickening of the ONL in the internal EDTRS after VN treatment in the eye without intra-operative foveal detachment of a selected patient (ID 6), whereas Fig. 4 showed a mild thinning of the central subfoveal ONL in the contralateral eye of the same patient, which had undergone intra-operative foveal detachment.

**Table 4:** Analysis of changes stratified according to intra-operative foveal detachment.

Variable	With intra-operative foveal detachment (macula OFF)* *N* = 5 eyes	Without intra-operative foveal detachment (macula ON)* *N* = 7 eyes	*P* value^†^
BCVA (ETDRS score)	− 0.20 ± 0.07	− 0.19 ± 0.07	0.459
Central foveal retinal thickness (µm)	− 5.2 ± 11.6	14.7 ± 19.8	0.054
Central foveal ONL thickness (µm)	− 1.8 ± 7.1	7.1 ± 5.9	**0.022**
ONL thickness of the internal ETDRS ring (µm)	2.2 ± 4.8	7.0 ± 5.8	0.127

*BCVA* best-corrected visual acuity, *ETDRS* early treatment diabetic retinopathy study, *ONL* outer nuclear layer.

*Data reported as mean ± standard deviation.

^†^*p*-values are obtained by generalized estimating equation regression models; statistically significant *p*-values are shown in bold.

## Discussion

The current study describes changes in visual function and retinal morphology, assessed by SD-OCT, in a small cohort of pediatric Italian patients with biallelic *RPE65*-associated IRDs treated with VN following approval of the gene therapy by the EMA. The current study sample included six pediatric patients treated at our Centre for whom volumetric SD-OCT scans of satisfactory quality were available at baseline. At the first post-treatment follow-up (i.e., about 45 days after the treatment of the first eye, corresponding to about 30 days after treatment of the second eye), we observed a significant improvement of BCVA (i.e., at least 1 ETDRS line) in all treated patients. These functional changes were associated with a significant increase in ONL thickness in the internal ETDRS ring (1–3 mm from the fovea). Moreover, after six months of follow-up, further improvements were observed in BCVA, while the increased ONL thickness remained stable.

BCVA changes after treatment with VN have been described both in clinical trial reports^4,5,8,9^, and in real-life case series^10–12^. Our findings are in line with these studies; however, quantitative morphological evaluations in patients treated with VN are limited, because in the phase 1 and 3 trials^4,5,8,9^ SD-OCT measurements were only used to evaluate the inclusion eligibility of the patients (i.e., central retinal thickness > 100 µm) and to assess the safety of the treatment (e.g., qualitative morphological changes, such as a macular hole). Changes in neurosensory retinal thickness at the fovea were investigated only in two recent case series^10,12^, which reported a small decrease in the majority of the analyzed pediatric eyes. However, the thickness of the ONL layer, which contains the nuclei of the cone and rod photoreceptors^16^, may be more representative of the effects of gene therapy on photoreceptor viability than whole-retina thickness. Indeed, the ONL thickness has been considered to be a relevant anatomical endpoint for ex vivo and animal studies of retinal degenerations^23–25^. Furthermore, SD-OCT imaging has enabled the direct measurement of the ONL thickness in animal models in order to monitor the natural history of disease and therapeutic intervention effects^13–15^. Despite the evidence from pre-clinical studies, only a few reports have analyzed ONL thickness in biallelic *RPE65*-related IRD after treatment with gene therapy^17,18,26^. In particular, Cideciyan et al. investigated ONL thickness in 11 patients with biallelic *RPE65*-associated Leber congenital amaurosis (LCA) treated with ocular gene therapy^26^, reporting a loss of ONL thickness in both treated and untreated retinas. However, the comparison of our findings with the literature cited above is limited by several differences in the methodology (e.g., surgical procedure and viral vector; software for measurement of ONL thickness) and study sample (e.g., patient age). In particular, Cideciyan et al.^26^ performed their ONL analysis along the vertical meridian and using a customized program, whereas we adopted a widely used approach based on commercial software to segment the ONL thickness and ETDRS grid evaluation. Lastly, Cideciyan et al. did not report BCVA data^26^.

Our study should be mainly compared with reports investigating the effects of VN treatment. A review of the literature using the search terms: “voretigene neparvovec” AND “optical coherence tomography” retrieved only one such report that analyzed ONL changes after VN treatment^12^. However, that study focused on measurements in the central foveal field and did not report any significant changes. On the contrary, we observed, for the first time in the literature, significant changes in the ONL, particularly in the perifoveal area, in a cohort of pediatric patients after VN treatment. Our findings suggest that treatment with VN halts photoreceptor degeneration, leading to a partial recovery of retinal morphology, particularly in the perifoveal area, i.e., 1–3 mm around the fovea, corresponding to 3.5°–7°, which has a high density of both rod and cones. These retinal changes are consistent with the BCVA improvement recorded in our treated patients. In fact, a previous study^27^ showed that BCVA obtained with a retinal region at an eccentricity of 5° is around 0.5 logMAR, which corresponds to the average BCVA achieved by our treated patients 6 months after treatment (0.50 ± 0.09 logMAR).

Consistently with the previous case series about treatments with VN^10^, we did not observe significant qualitative changes in the EZ band. Furthermore, these findings are in line with a recent study^28^ suggesting that the absence or presence of reflectivity in the EZ band does not reliably predict the degree of residual photoreceptor structure. In line with recent real-life case series^10,12^, we observed the development of multifocal retinal atrophy after VN treatment in some patients. In particular, the new areas of retinal atrophy were observed outside vascular arcades in both eyes of three of our treated patients, with no impact on macular function or morphology.

Finally, we explored the impact of intra-operative foveal detachment during the surgical procedure on visual acuity and retinal morphology. BCVA improved regardless of whether the fovea was detached or not. In contrast, we observed significant differences in the foveal ONL thickness between the two subgroups. In particular, while central ONL thickness mildly decreased in eyes undergoing intra-operative foveal detachment, it increased in eyes without foveal detachment. However, the differences in the foveal thickness of all neurosensory layers between the two subgroups were not statistically significant, although a similar trend was observed (i.e., a reduction in eyes undergoing intra-operative foveal detachment, an increase in eyes without). Furthermore, EZ band grading remained unchanged regardless of whether or not the fovea was detached.

The absence of significant differences in BCVA changes, in central foveal thickness and in EZ band grading between VN-treated eyes with and without intra-operative foveal detachment was reported by Sengillo et al.^10^. However, the authors did not analyze ONL thickness. On the contrary, the report of the phase 1 trial using a different rAAV2-RPE65 vector showed a short-term thinning of the ONL in most eyes with foveal detachment, cautioning against detaching the fovea^17^. Although further evidence is needed to settle the debate about intra-operative foveal detachment, our findings suggest that intra-operative foveal detachment may not be useful in achieving the therapeutic effect of VN.

The current study has some limitations, particularly related to the relatively low sample size, due to the challenges in obtaining SD-OCT scans of satisfactory quality in pediatric patients with poor cooperation, nystagmus, poor vision and inability to fixate. For this reason, we chose to include only patients with SD-OCT scans of satisfactory quality before treatment to perform a first preliminary analysis of ONL changes after VN treatment, even if in a small study sample size. Therefore, our results must be interpreted in light of this possible selection bias. Moreover, the current study explored only short-term changes, and further studies in larger samples and over longer follow-up durations are required to confirm our results.

## Conclusions

We evaluated morphological changes after VN administration, including the estimation of ONL thickness, in relationship to visual function. Our findings show, for the first time, a thickening of the ONL layer in the perifoveal ring (1–3 mm from the fovea) after VN treatment in a cohort of pediatric subjects. This suggests that the improvement of BCVA after VN treatment is related to a partial recovery of retinal morphology in the perifoveal ring, an area with a significant density of cone and rod photoreceptors. In this regard, ONL thickness could be explored as a surrogate marker of treatment effects, particularly in non-verbal children. Finally, intra-operative foveal detachment was not associated with a higher function gain in terms of BCVA, but with a mild thinning of foveal ONL after treatment. Therefore, caution should be taken to avoid intra-operative foveal detachment during VN surgery, given that such a procedure does not contribute to better treatment effects.

## Data Availability

The datasets generated during or analyzed during the current study are available from the corresponding author on reasonable request.
